# Safety or Autonomy in Dementia Care at Home, a Qualitative Study on Family Caregivers’ Experiences

**DOI:** 10.1093/geroni/igaa057.1406

**Published:** 2020-12-16

**Authors:** Vincent Moermans, Michel Bleijlevens, Hilde Verbeek, Koen Milisen, Bernadette Dierckx de Casterlé, Jan Hamers

**Affiliations:** 1 Maastricht University, Riemst, Limburg, Belgium; 2 Maastricht University, Maastricht, Limburg, Netherlands; 3 KU Leuven, Leuven, Belgium

## Abstract

Persons with Dementia (PwD) living in their own home become increasingly dependent on support from especially family caregivers who often are confronted with dilemmas. Family caregivers feel responsible for PwD and predominantly want to ensure their safety, whereas PwD wants to engage in meaningful activity and make decisions autonomously. This study aims to identify how family caregivers caring for PwD at home deal with these dilemmas. Using a grounded theory approach, this study conducted in-depth interviews with 8 family caregivers. Results show that dealing with the dilemma of safety versus autonomy is considered as a calculated risk. By timely entering into dialogue with their loved one, family caregivers try to create a safe environment taking into account the needs and preferences of PwD. However, when situations evolve to such an extent that family caregivers think unsafety will occur, they take over control and overrule the autonomy of the PwD. This often results in resistance to care in PwD. Dealing with the dilemma is a continuous and exhausting learning process due to the ever-changing behaviour of PwD. Family caregivers said that they are in need of support of professional caregivers, but that this need is met insufficiently. As a result, family caregivers have to rely mostly on their own intuition and earlier experiences. The conclusion is that family caregivers experience serious dilemmas in the care of PwD living at home. Professional support in making adequate decisions related to safety and autonomy is warranted.

